# Comment on Sundseth et al. Global Sources and Pathways of Mercury in the Context of Human Health. *Int. J. Environ. Res. Public Health* 2017, 14, 105

**DOI:** 10.3390/ijerph14050481

**Published:** 2017-05-05

**Authors:** S. M. J. Mortazavi, Ghazal Mortazavi, Maryam Paknahad

**Affiliations:** 1 Diagnostic Imaging Center, Fox Chase Cancer Center, Philadelphia, PA 19111, USA; s.m.javad.mortazavi@fccc.edu; 2 Ionizing and Non-ionizing Radiation Protection Research Center (INIRPRC), Shiraz University of Medical Sciences, Shiraz 7134845794, Iran; qaz.mortazavi@gmail.com; 3 Oral and Maxillofacial Radiology Department, School of Dentistry, Shiraz University of Medical Sciences, Shiraz 713451836, Iran

**Keywords:** mercury, global sources, dental amalgam, electromagnetic fields, non-ionizing radiation

It was with great interest that we read the article by Sundseth et al. [1] entitled Global Sources and Pathways of Mercury in the Context of Human Health which is published in the *Int. J. Environ. Res. Public Health* **2017**, *14*, 105; doi:10.3390/ijerph14010105. In this article, the authors reviewed the current scientific knowledge on global mercury releases into the atmosphere, global atmospheric transport and deposition, and the linkage between environmental contamination and potential impacts on human health. Unfortunately, the authors have ignored the effect of modern life and advanced technologies on the health effects of mercury. Humans are now immersed in an ocean of electromagnetic fields (EMFs). Advances in technologies such as telecommunication has led to an exponential rise in human exposure to EMFs emitted from wireless devices such as mobile phones, cordless phones and Wi-Fi routers. Moreover, the number of mobile base stations in our living environment is increasing rapidly. We have previously discussed in detail that when dental amalgam restorations are exposed to EMFs produced by magnetic resonance imaging (MRI) or other sources such as mobile phones or Wi-Fi routers, a significant rise in the level of mercury release from dental amalgam restorations can be observed [2,3]. It is worth mentioning that the findings obtained in our studies on EMF-induced accelerated mercury release are further supported by microleakage studies [4,5]. Furthermore, there are reports which show that even exposure to ionizing radiations such as x-rays can accelerate the release of mercury from dental amalgam fillings [6]. This issue has previously been addressed in a review article by Mortazavi and Mortazavi [7]. In this light, a shortcoming of the article authored by [1] comes from this point that these researchers have not considered the impact of modern technologies on the challenging issue of the effect of low levels of mercury on human health.
